# Yerba Mate Modulates Tumor Cells Functions Involved in Metastasis in Breast Cancer Models

**DOI:** 10.3389/fphar.2021.750197

**Published:** 2021-11-11

**Authors:** Garcia-Lazaro Rocio Soledad, Caligiuri Lorena Gisel, Lorenzo Norailys, Lamdan Humberto, Alonso Daniel Fernando, Farina Hernan Gabriel

**Affiliations:** Molecular and Translational Oncology Center, Science and Technology Department, National University of Quilmes, Buenos Aires, Argentina

**Keywords:** breast cancer, Yerba mate, polyphenols, tumor progression, metastasis

## Abstract

Breast cancer (BC) is the most frequent cancer in women and tumor metastasis is a major cause of cancer-related deaths. Our aim was to evaluate anti-metastatic properties of yerba mate extract (YMe) in BC models. 4T1, F3II, MCF-7, and MDA-MB231 cell lines were used to perform *in vitro* assays. The F3II syngeneic mammary carcinoma model in BALB/c mice was used to evaluate tumor progression, BC metastasis and survival. Cells were inoculated subcutaneously into the flank for the heterotopic model and into the mammary fat pad for the orthotopic model. YMe was administered p.o. in a dose of 1.6 g/kg/day. *In vitro* YMe inhibited cell proliferation and reduced tumor cell adhesion, migration and invasion. These biological effects were cell-line dependent. *In vivo* YMe reduced tumor metastasis and increased mice survival in both models. Our preclinical results suggest that YMe could modulate tumor progression and metastasis in BC models.

## Introduction

BC is the most prevalent female cancer worldwide along with cervical cancer and both are considered the leading causes of death from cancer in women (Torre et al., 2016). Negative outcomes for patients with BC and the clinical complications associated with this pathology are largely due to the development of metastases. On advancement of the disease, tumor cells acquire some capabilities that allow them to leave the primary tumor and colonize a secondary organ (Hanahan and Weinberg 2011; Pillar et al., 2018).

Tumor cells can colonize a new tissue and it shows an organ-specific pattern of metastasis. In BC disease, bones and lungs are the most frequent sites of metastasis (Krishnan et al., 2006; Yates et al., 2017). Lung metastases show no symptoms, until the lungs have a high amount of tumor metastasis, so clinical prognosis and treatment are significantly compromised. It is demonstrated that in BC, the 5-years overall survival is 96% for localized disease, in contrast with 21% for patients with metastatic disease (Shi et al., 2020). Although clinical management has progressed substantially over the past years, at present, there is no cure currently available for metastatic BC. New approaches for treatment of BC metastasis could be very useful for cancer therapy.

Although products from the plant kingdom have been used since ancient times, in the search of new therapeutic agents for BC metastasis, natural compounds have gained great importance in recent years (Cragg and Pezzuto 2016) due to many reasons: available therapies are, in some cases, inefficient or have adverse side effects. In addition, they are expensive. The uses of these natural compounds range from homemade medicine (herbal tea), pharmaceutical preparations (crude extract or fractions of the crude extract enriched with some bioactive compound such as fluid or powder, capsules or pills) or as a drug (in the case that after successive extractions a pure compound is isolated) (Rates 2001).

Plants contain phytochemicals, which are secondary metabolites with assigned functions such as defense, pollinator attraction, support and protection against UV radiation and various pathogens among others (Kapinova et al., 2018). A group of these phytochemicals, polyphenols, which are present in high amounts in many plants are reported to exhibit many biologically significant functions. Numerous studies indicate that polyphenols have an antioxidant capacity (Scalbert et al., 2007; Maqsood et al., 2014) and anti-inflammatory activity (Yoon and Baek, 2005; Zhang and Tsao, 2016). Furthermore, these compounds prevent and reduce the risk of contracting certain chronic diseases such as cardiovascular diseases (Weisburger 2001; Marventano et al., 2016), type 2 diabetes mellitus (Viguiliouk et al., 2014), neurodegenerative diseases (Liu et al., 2017) and different types of cancer (Niedzwiecki et al., 2016; Kapinova et al., 2018).

As mentioned previously, many studies have demonstrated that polyphenols have a broad range of effects on the human body. In what concerns cancer, polyphenols have demonstrated anti-tumor activity. They act on several molecular targets and the anti-tumor effects include: inhibition of cell growth, induction of apoptosis, reduction of cancer invasion and angiogenesis (Kou et al., 2016; Dai et al., 2017).

Yerba mate (YM), under the botanical name *Ilex paraguariensis*, is a plant native to South America which grows in Argentina, Paraguay, Uruguay, and Brazil (Bastos et al., 2007; Heck and De Mejia 2007). YM is an excellent source of polyphenols, specifically: caffeoyl derivatives (caffeic acid, chlorogenic acid, 3, 4-dicaffeoylquinic acid, 3, 5-dicaffeoylquinic acid, and 4, 5-dicaffeoylquinic acid); these components are responsible for the antioxidant activity attributed to YM (Filip et al., 2000). YM is also abundant in xanthines and saponins. The most abundant xanthines in this vegetable are theophylline, theobromine, and caffeine. Regarding saponins, it is believed that these components are what give the characteristic flavor to the infusion. YM also contains other components such as minerals and vitamins (Heck and De Mejia 2007).

YM is endowed with several biological properties, which many authors attribute to polyphenols (Bastos et al., 2007). It has been demonstrated that YM has antioxidant capacity (Filip et al., 2000), anti-inflammatory properties (Alves et al., 2019), anti-obesity effect (Ko and Auyeung 2013), cardiovascular protective effect (de Veiga et al., 2018), and anti-tumoral effects (Amigo-Benavent et al., 2017). There are studies about the anti-cancer properties of YM and its polyphenols. Yamagata and co-workers have demonstrated that chlorogenic acid (the main polyphenol in YM) affects the expression of apoptosis-related genes in A549 human lung cancer cells (Yamagata et al., 2018). Through an *in vivo* assay, Kang et al. demonstrated that chlorogenic acid reduced colon cancer metastasis (Kang et al., 2011). Wang and co-workers demonstrated that YM extract affects the viability and proliferation of different tumor cell lines (Caco-2, A549, OE-33, and T24) (Amigo-Benavent et al., 2017). Our group reported that YMe showed a noticeable anti-proliferative activity against CT26 and COLO-205 tumor cell lines and modulated cell adhesion, migration, and invasion. In addition, YMe exerted *in vivo* antiangiogenic and anti-tumor effects (Garcia-Lazaro et al., 2020). Ronco et al. conducted a case-control study and reported an inverse association between high “mate” intake and breast cancer risk (Ronco et al., 2016).

In view of the high incidence of BC cancer in women and knowing that the development of distant metastases is a major cause of death from BC, we set out to evaluate how an YMe acts on the different steps of the metastatic cascade using *in vitro* and *in vivo* models. The main overall goals of this work have been on the one hand, to investigate whether a YMe can modulate certain events like: cell proliferation, adhesion, migration and invasion using *in vitro* assays and on the other hand, to study how the extract regulates clinically relevant parameters such as progression, survival and development of metastasis using both orthotopic and heterotopic *in vivo* BC models.

## Materials and Methods

### Preparation of YMe

YMe was generated by aqueous extraction as describe in our previous work (Garcia-Lazaro et al., 2020). Briefly, YM leaves were macerated and then, the mixture was concentrated until 25° Brix, using maltodextrin (MD). Immediately, the resulting solution was incorporated into a pilot scale spray dryer (Galaxie, model 1,612). The powder was collected and stored in polyethylene bags at room temperature and protected from light. Prior to use, the extracts were dissolved in double-distilled water (ddH_2_O) and filtered with a 0.22 µm. Extracts were standardized to total phenolic content, antioxidant activity, and Chlorogenic Acid content.

### Chemical Composition

Protein, moisture, lipid, total ash, and dietary fiber were determined using the Association of Official Analytical Chemists (AOAC) methods (Horwitz and Latimer, 2006). The total carbohydrate content was calculated as the difference between 100 and the sum of the percentages of moisture, protein, lipid, ash and dietary fiber. Sugars were determined using AOAC methods, and are the sum of individual monosaccharides (glucose and fructose) and disaccharides (sucrose and maltose). Energy values were obtained by applying factor 4–4–9 kcal/g for protein, carbohydrate, and lipid, respectively (Bertë et al., 2011).

### Cells and Cell Culture Conditions

The cells were cultured at 37°C in a humidified atmosphere containing 5% CO_2_. 4T1 (ATCC: CRL-2539) from mouse mammary tumor was grown in Roswell Park Memorial Institute (RPMI) 1,640 medium (Life Technologies, United States). A sarcomatoid mammary carcinoma cell line F3II is a highly invasive and metastatic variant established from a clone of a spontaneous, hormone-independent BALB/c mouse mammary tumor (Alonso et al., 1996), MCF-7 (ATCC: HTB-22, human breast adenocarcinoma) ER/PR + human BC cells and MDA-MB 231 (ATCC: HTB-26, human breast adenocarcinoma) a triple-negative human BC cells were grown in high-glucose Dulbecco’s Modified Eagle’s Medium (DMEM) (Life Technologies, United States). The characteristics of cell lines are shown in Table 1. All cultures contained 10% fetal bovine serum (FBS, Gibco, United States) and 40 μg/ml gentamicin (Fada Pharma, Argentina). In addition, cells lines were routinely tested to rule out *Mycoplasma* infection of cells.

**TABLE 1:** Cell lines characterization.

Cell line	Status of hormone receptors	Her2 expression	Metastatic/invasive capacities	Tumorigenic
F3II	ER + PR−	HER−	+++	Yes, forms tumors and metastasis in BALB/c mice
4T1	ER− PR−	HER−	+++	Yes, forms tumors and metastasis in BALB/c mice
MCF-7	ER + PR+	HER+	+	Cells are not tumorigenic in immunocompromised mice unless estrogen supplementation is carried out and yet they express a less aggressive phenotype
MDA-MB 231	ER + PR−	HER−	++	Yes, in ALS treated BALB/c mice, forms poorly differentiated adenocarcinoma (grade III)
				Yes, in nude mice, forms poorly differentiated adenocarcinoma (grade III)

### Cell Proliferation Assay

4T1, F3II, MCF-7, and MDA-MB 231 BC cells were seeded into 96-well plates at 2.5×10^3^ cells per well and incubated for 24 and 72 h at 37°C, 5% CO_2_. YMe concentrations ranging from 0.03 to 2.5 mg/ml were added to the wells in complete medium. After incubation, the medium was removed and the plates were washed with PBS. Then, attached cells were fixed with methanol for 15 min and stained with 0.5% crystal violet for another 15 min. The excess dye was removed by washing with rinse water. The dye in the cells was dissolved in methanol-acetic solution (10%/5%, V/V) and the absorbance was measured at 595 nm in a 96-well plate reader (ASYS Hitech Gmbh, Austria). Each condition was assayed sextuplicate in three different experiments, and SD was determined. Inhibitory concentration 50 (IC_50_) values were calculated from the growth curves for 72 h.

### Terminal Deoxynucleotidyl-Transferase-Mediated dUTP Nick End Labeling (TUNEL) Assay

Detection of apoptosis in all samples were provided using a TUNEL (Terminal deoxynucleotidyl transferase dUTP nick end labeling) assay Kit (Promega) according to manufacturer’s protocol for detection of DNA fragmentation, a prominent hallmark of apoptosis. Briefly, F3II cells growing in glass coverslips were treated with 0.15 mg/ml of YMe. The cells treated with PBS were considered as negative control and the cells treated with Camptotecin (10 µM) for 4 h were considered as positive control. After 24 h of treatment, TUNEL assay was carried out. F3II cells were fixed for 10 min at room temperature in a solution containing 4% (W/V) of paraformaldehyde in PBS (pH 7.4). Then, cells were washed 3 times with PBS. Subsequently, cells were permeabilized with 0.2% Triton X-100 for 5 min on ice. After washing with PBS, cells were incubated with equilibration buffer for 10 min at room temperature. TUNEL reaction mixture (50 µL) containing the TdT enzyme was added to the cells. After that, the cells were incubated in a humidified box in the dark for 1 h at 37°C. Then, the cells were washing with PBS 3 times. To detect the nuclei, the samples were counterstained using 4, 6-diamidino-2-phenylindole (DAPI) for 2 min at room temperature in the dark and then washed with PBS 3 times. The TUNEL stained sections were viewed under fluorescence microscope (Cytation 5—Biotek) at 2 different wavelengths for fluorescein isothiocyanate (FITC) and DAPI respectively, and images of 10 randomly selected fields were captured at 10X magnification for each slide. The apoptotic index (AI) was then calculated by the following formula: AI= (Number of TUNEL positive cells/Total number of cells) x 100.

### Adhesion Assay

Cell adhesion assay was carried out by a colorimetric method based on staining cells with the dye crystal violet. Briefly, 4T1, F3II, MCF-7, and MDA-MB 231 BC cells in 200 µL medium with FBS were seeded into 96-well plate at 4×10^4^ cells/well. The cells were treated with YMe with concentrations ranging from 0.03 to 2.5 mg/ml for 2 h. The plates were incubated at 37°C, 5% CO_2._ After that, PBS was added to each well and then aspirated to remove non-adhered cells. Then the cells were fixed and stained with 0.1% crystal violet, 20% methanol in PBS for 20 min. The excess dye was removed by washing and dye in the cells was dissolved in methanol-acetic solution (10%/5%, V/V). The absorbance was measured at 595 nm in a 96-well plate reader (ASYS HitechGmbh, Austria).

### Cell Migration Wound Healing Assay

4T1, F3II, MCF-7, and MDA-MB 231 BC cells were seeded at a density of 1×10^5^ cells/well in a 6-well plate. When cells reached 80% confluence, wounds were made in the cell monolayer with a pipette tip and photographed (time 0) at 40X using an inverted microscopy. Cells were incubated during 12 h in presence of IC_50_ of YMe. After that, cells were washed with PBS, fixed with methanol and stained with 0.5% crystal violet. Then, cells were photographed again at 3 randomly selected sites per well. Photographs were taken using a camera connected to the inverted microscope (Nikon, NIS elements software), and invasion area was quantified using ImageJ software. Data are expressed as mean ± SD. The treatments were carried out in triplicate and each experiment was repeated 3 times independently.

### Transwell Invasion Assay

4T1 and F3II BC cells (2×10^5^) were plated in 100 µL of medium without FBS in the upper chamber of 8 µm Transwells (Costar Inc.), coated with 100 µL of 0.1 mg/ml Matrigel. Cells were incubated during 24 h in presence of IC_50_ of YMe. Complete medium (with FBS as chemoattractant) was placed in the lower chamber. After the incubation period, cells that remained in the upper chamber were removed using a cotton swab. Cells adhered in the transwell lower chamber were washed with PBS, fixed and stained with 0.1% crystal violet, 20% methanol in PBS for 20 min. Cells that invaded and migrated to the lower chamber were photographed (Nikon, NIS elements software) and quantified. The number of cells were evaluated in five random areas using a phase contrast inverted microscope (40X magnification).

### Animals

Four-week old pathogen-free female BALB/c mice were obtained from La Plata University. The mice were housed in 12 h of light and dark cycle. Food and water were provided ad libitum, and general health status of the animals was monitored daily. Animals with an average weight of 20 g were used.

### Orthotopic BC Model

Mice were randomly assigned into 2 groups (n per group = 11). Control group drank MD, the excipient of extract and treated group drank YMe 10 mg/ml. The YMe was administered to BALB/c mice in a dose of 1.6 g/kg/day via the drinking water before (1 month) and after the inoculation of F3II tumor cells. Mouse mammary cancer model was established through subcutaneously inoculating 1 × 10^5^ F3II cells into the left fourth mammary fat pad (MFP) in mice.

### Heterotopic BC Model

Mice were randomly assigned into 2 groups (n per group = 6). Control group drank MD and treated group drank YMe 10 mg/ml. The YMe was administered to BALB/c mice via the drinking water before (1 month) and after the inoculation of F3II tumor cells. The animals were inoculated with 5×10^4^ cells/mice on the right flank of female BALB/c mice.

### Tumor Growth and Metastasis

1 week after inoculation of F3II cells, tumors were measured 3 times per week using a digital caliper. The greatest longitudinal diameter (length) and the greatest transverse diameter (width) were measured and volume was calculated using the following formula: tumor volume (mm^3^) = ½ (length × width^2^). When the tumor volume reached about 1800 mm^3^, mice were sacrificed by cervical dislocation due to ethical considerations and tumors were resected from mice. Tumors were weighed, and the mean tumor weight was calculated. For spontaneous metastasis, lungs were collected and fixed in Bouin’s solution. The number of metastasis nodules in lung surface was manually counted using a dissecting microscope.

### Histopathological Studies

At the end points, mice were necropsied and the tumors and lungs were harvested, fixed in 10% formaldehyde (Anedra) and embedded in paraffin, cut into 5-mm sections, and stained with hematoxylin and eosin (H&E). Metastatic tumor nodules present on lungs were quantified by counting 3 sections per lung sample. Images were taken by an inverted microscope (Cytation 5—BioTek).

### Animal Ethics Statement

All animal protocols have been carried out in accordance with the Guide for the Care and Use of Laboratory Animals as adopted by the U.S. National Institutes of Health. Protocols were approved by our institutional Animal Care Committee UNQUI-CICUAL (Resolution CD CyT Nº075/14).

### Statistical Analysis

All data analyses were performed using GraphPad Prism version 6.00 (GraphPad Software, San Diego California, United States). Samples were examined for normality with Kolmogorov–Smirnov test. Results were expressed as mean ± standard deviation (SD) or standard error of mean (SEM), and differences were analyzed with Student’s t test or ANOVA with a Tukey’s post-test, accordingly. Survival curves were plotted according to the Kaplan–Meier method. Statistical significance was calculated using log-rank test. *p* < 0.05 was considered statistically significant.

## Results

### Chemical Composition of YMe

The chemical composition of spray-dried extract is shown in Table 2. Considering the nutritional components of our extract in addition to the high antioxidant capacity exerted by their polyphenolic compounds and the bioactivities against colon tumor cells (Garcia-Lazaro et al., 2020), we became interested in studying the anti-tumor effects of YMe in different *in vitro* and *in vivo* breast cancer models.

**TABLE 2:** Chemical composition in the spray-dried extract.

Component	Spray-dried extract
Total carbohydrates (gr)	77.30 ± 0.42
Fructose	1.40 gr.%
Glucose	3.0 gr.%
Sucrose	5.30 gr.%
Maltose	2.10 gr.%
Protein (gr)	9.40 ± 0.03
Lipid (gr)	0.00 ± 0.00
Total ash (gr)	8.40 ± 0.47
Dietary fiber (gr)	1.10 ± 0.15
Humidity (gr)	2.10 ± 0.20
Calories (kcal/100 gr)	347 ± 1.42
Total polyphenols (mg GAE/mL) *	702 ± 29.3
Chlorogenic acid (mg/gr) *	66.30 ± 0.05
Rutin (mg/gr) *	6.783 ± 0.05
Gallic Acid (mg/gr) *	6.665 ± 0.32
Caffeic acid (mg/gr) *	0.533 ± 0.04
Quercetin (mg/gr) *	0.229 ± 0.02

The values are mean ±SD (n = 3); *Garcia-Lazaro et al. (2020).

### Anti-Proliferative Effect of YMe on Tumor Cell Lines

**FIGURE 1:**
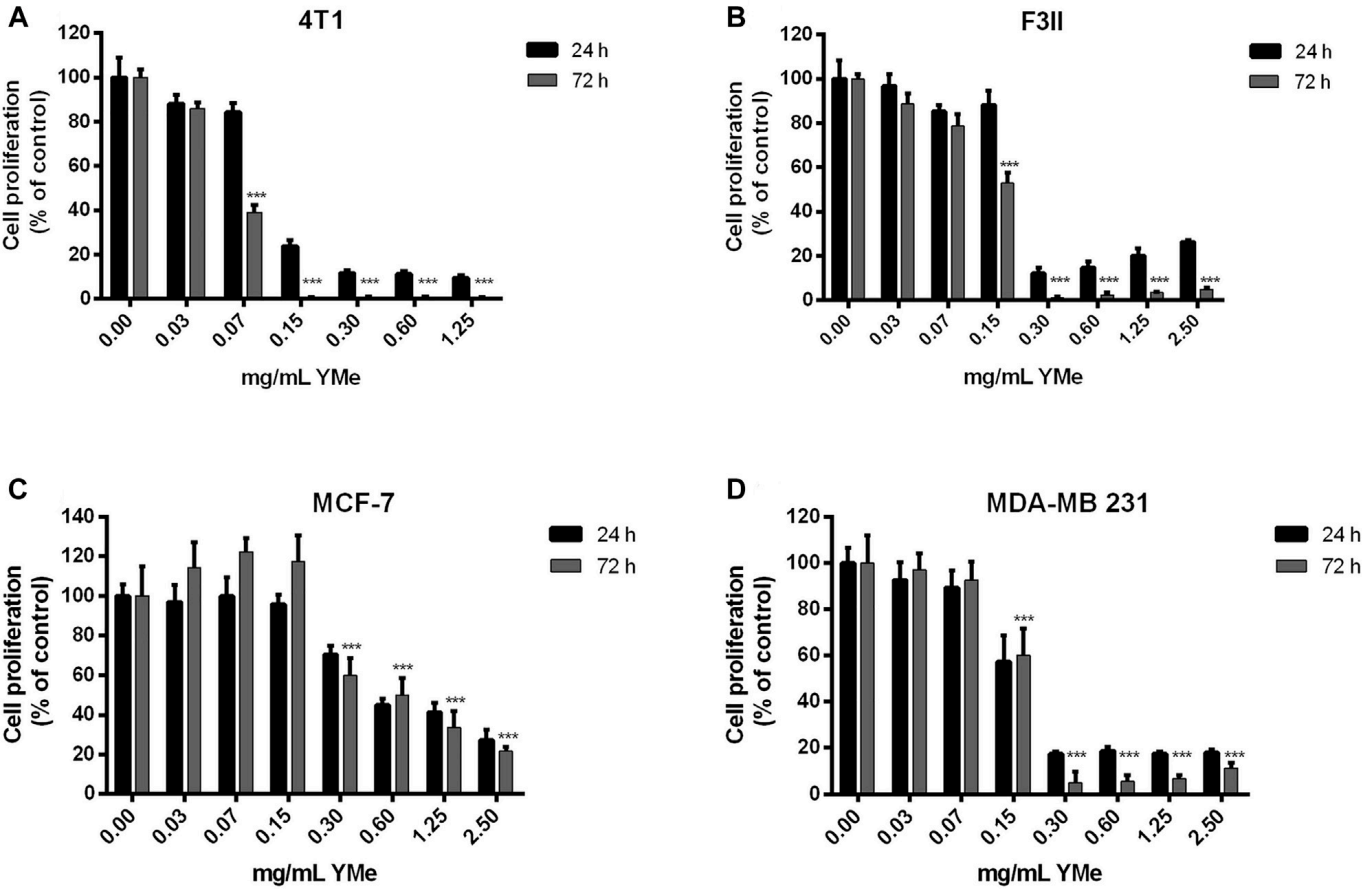
Anti-proliferative effect of YMe. Breast cancer cell lines, 4T1 **(A)**, F3II **(B)**, MCF-7 **(C)**, and MDA-MB 231 **(D)** were treated with increasing concentrations of YMe for 24 and 72 h. Cell proliferation was determined by a colorimetric method. Each point represents the average of six independent measurements, each done in triplicate with the standard deviation. Inhibitory concentration 50 (IC_50_) values were calculated from the growth curves for 72 h. Statistical analysis was done using ANOVA and Tukey post-test; ***p < 0.001 *vs.* control.

To study the sensitivity of BC cells to YMe, we first evaluated its effect on cell proliferation of murine and human BC cell lines. As shown in Figure 1, 4T1 (Figure 1A), F3II (Figure 1B), MCF-7 (Figure 1C), and MDA-MB 231 (Figure 1D) cells were treated with different concentrations of the extract ranging from 0.03 to 2.50 mg/ml for 24 and 72 h. YMe showed a statistically significant decrease in growth of murine and humane cells *in vitro* between the control and all treated cells. IC_50_ values were calculated from the growth curves for 72 h. The IC_50_ values obtained were 0.06 mg/ml to 4T1, 0.15 mg/ml to F3II, 0.6 mg/ml to MCF-7 and 0.15 mg/ml to MDA-MB 231. These values had no cytotoxic effects when assayed for 24 h on semi-confluent monolayers. Since Maltodextrin was used as a carrier agent during spray-drying process, we evaluated whether this vehicle modulates cell proliferation. We observed that cell proliferation was not affected when the tumor cells were treated with this excipient (data not shown).

**FIGURE 2:**
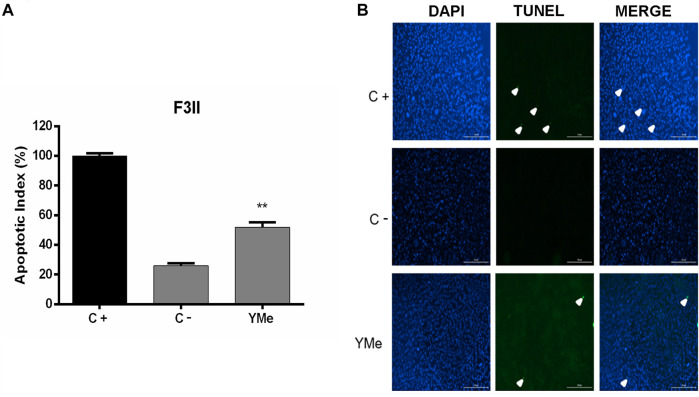
Effect of YMe on apoptosis of F3II cells by TUNEL method. Terminal deoxynucleotidyl transferase nick end-labeling (TUNEL) was used to detect apoptotic cells. The cells treated with PBS were considered as negative control (C-) and cells treated with Camptotecin (10 µM) for 4 h were considered as positive control (C+). After 24 h of treatment, TUNEL assay was carried out. Assay was performed on cells growing in glass cover slips and then stained as described. **(A)** An increase in TUNEL staining in F3II cells treated with YMe was observed. The apoptotic index (AI) was calculated by the following formula: AI= (Number of TUNEL-positive cells/Total number of cells) x 100. **(B)** Cells stained with TUNEL (green) and counterstained with DAPI (blue) respectively. Blue and green stain in the merged image represents the TUNEL-positive apoptotic cells. Scale bars = 200 µm.

To understand the effect of YMe on cell proliferation, the mechanism of cellular apoptosis was investigated. A TUNEL assay was conducted to evaluate the cell death following treatment with the IC_50_ concentration of YMe for 24 h. As shown in Figure 2A the AI was significantly elevated in F3II cells treated with YMe compared to the untreated cells (*p < 0.05). Cells were stained with TUNEL (green) and counterstained with DAPI (blue) respectively. Blue and green stains in the merged image represent the TUNEL-positive apoptotic cells.

### Effects of YMe on Cell Adhesion

**FIGURE 3:**
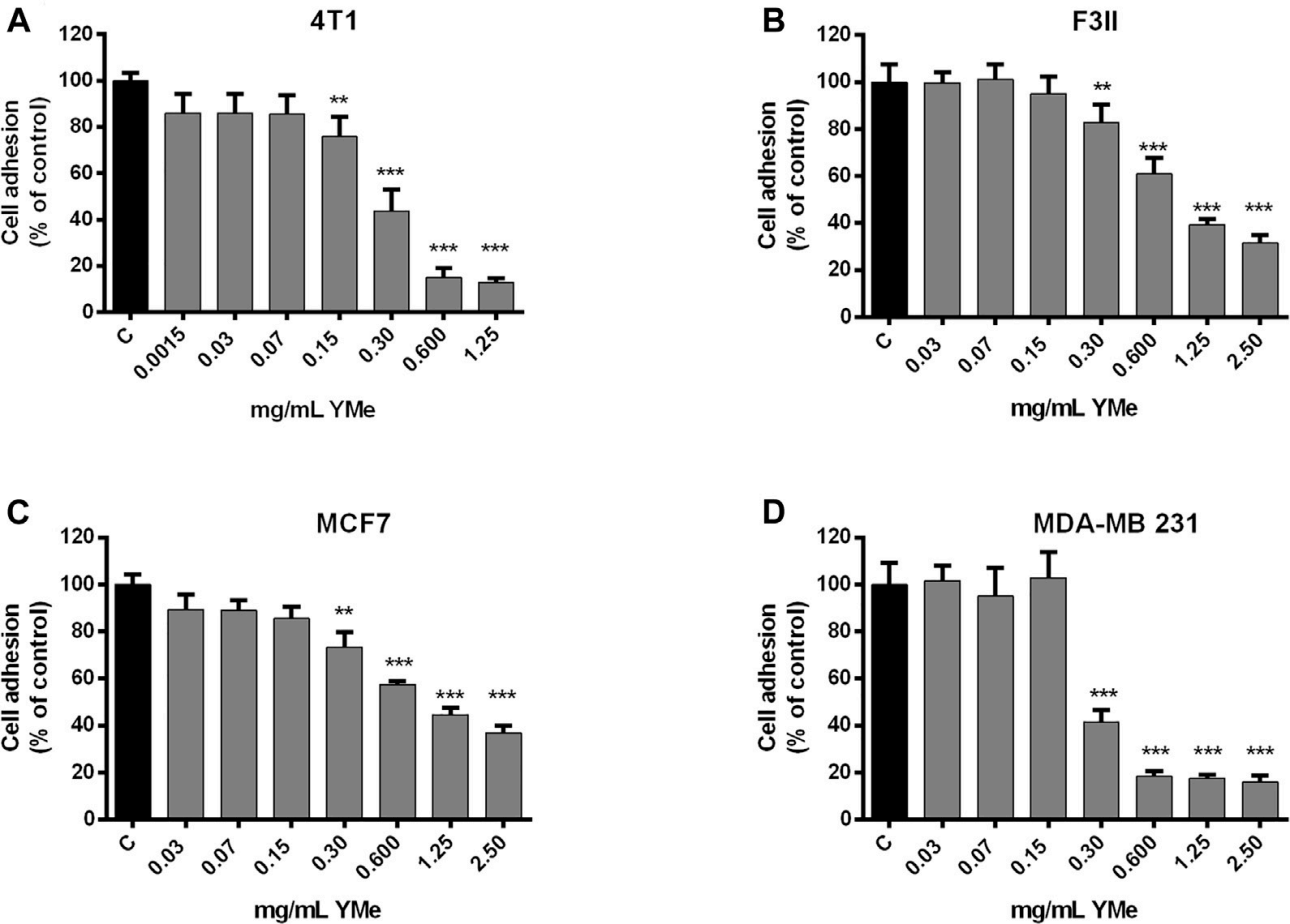
Inhibitory effect of YMe on cell adhesion. 4T1, F3II, MCF-7 and MDA-MB 231 (**A,B,C,D** respectively) BC cell lines were treated with increasing concentrations of YMe for 2 h. After washing, adherent cells were stained with crystal violet, solubilized in methanol-acetic solution (10%/5%, V/V) and the absorbance was measured at 595 nm. Data represent the means ± SD (n = 6) and were expressed as percentage of adhesion respect to the control. Each point represents the average of six independent measurements, each done in triplicate with the standard deviation. Statistical analysis was done using ANOVA and Tukey post-test; **p < 0.01, ***p < 0.001 *vs.* control.

We investigated whether YMe can act through the blocking of cell attachment using a cell adhesion assay. The cells were seeded and treated with increasing concentrations of YMe. Cell adhesion of 4T1 (Figure 3A), F3II (Figure 3B), MCF-7 (Figure 3C), and MDA-MB 231 (Figure 3D) cells lines decreased significantly in a dose-dependent manner in response to treatment with YMe (**p < 0.01, ***p < 0.001 *vs.* control).

### Effects of YMe on Cell Migration and Invasion

**FIGURE 4:**
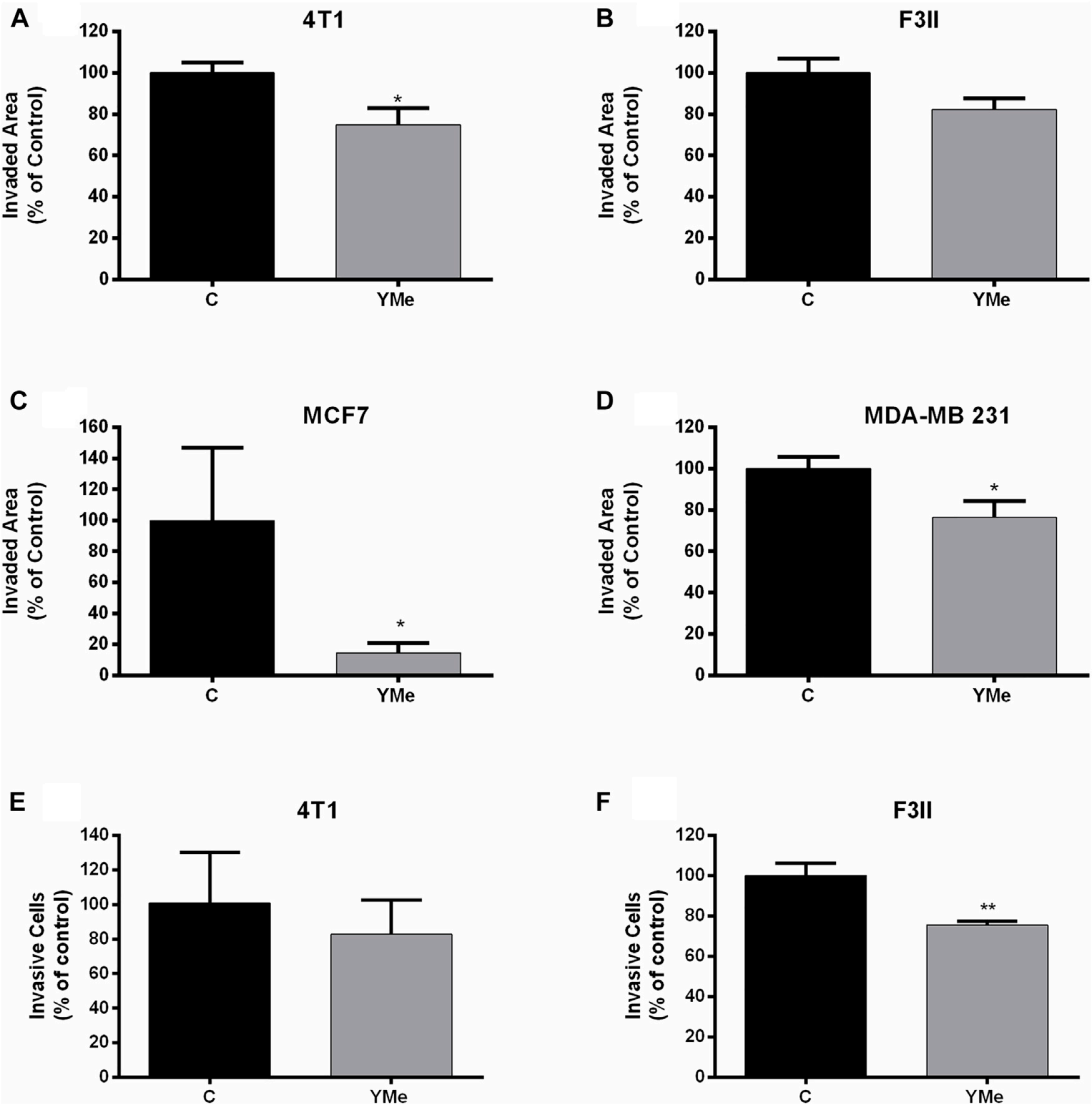
Effect of YMe on cell migration and invasion. YMe inhibited the cell migration of 4T1, F3II, MCF-7 and MDA. MB 231 (**A,B,C,D** respectively). BC cell lines were cultured to 85% confluence in a 6-well plate and the cell layer was scratched with a yellow micropipette tip. Cells were incubated in the presence of YMe for 12 h. Before and after incubation, images were photographed using an inverted microscope (Nikon, NIS elements software) (40X magnification). Quantification of wound closure distance was made. Values are expressed as mean ± SEM. *p < 0.05, Student’s t test. To examine the effect of YMe on the ability of cells to move in a chemoattractant gradient, a transwell invasion assay was used. 4T1 and F3II cells (**E,F** respectively) were suspended in serum-free medium and seeded in the upper chamber of transwells. Following incubation with YMe for 24 h, invaded cells were stained with crystal violet and counted under an inverted microscope (Nikon, NIS elements software) (40X magnification). Data are expressed as the mean ± SEM (n = 3). Three independent experiments were performed. **p < 0.01 *vs*. control cells, Student’s t test.

The effect of YMe on cell motility was evaluated using wound healing assays using a non-cytotoxic concentration of the extract. After scratching confluent monolayers of 4T1, F3II, MCF-7, and MDA-MB 231 cells to create a wound-like gap, the cells were treated with IC_50_ of the extract. As shown in Figure 4, after 12 h of treatment with the extract, cell motility was inhibited by YM. The wounds of treated groups remained cracked with some differences. The treatment with YMe reduced the motility of 4T1 (Figure 4A) and MDA-MB 231 (Figure 4D) by 25%, F3II cell line by 18% (Figure 4B) and MCF-7 by 82% (Figure 4C). The motility reduction in the F3II cell line was not statistically significant.

A transwell assay was used to evaluate whether YMe affects the invasion of BC cells on the ability of cells to migrate in a chemoattractant gradient. As shown in Figures 4E,F, the invasiveness of 4T1 and F3II tumor cells treated with YMe was decreased in comparison with the control group. This reduction was statistically significant in the F3II cell line (**p < 0.01 vs. control cells) but no in the 4T1 cell line.

### *In vivo* Anti-Tumor Effect of YMe on Both Models

**FIGURE 5:**
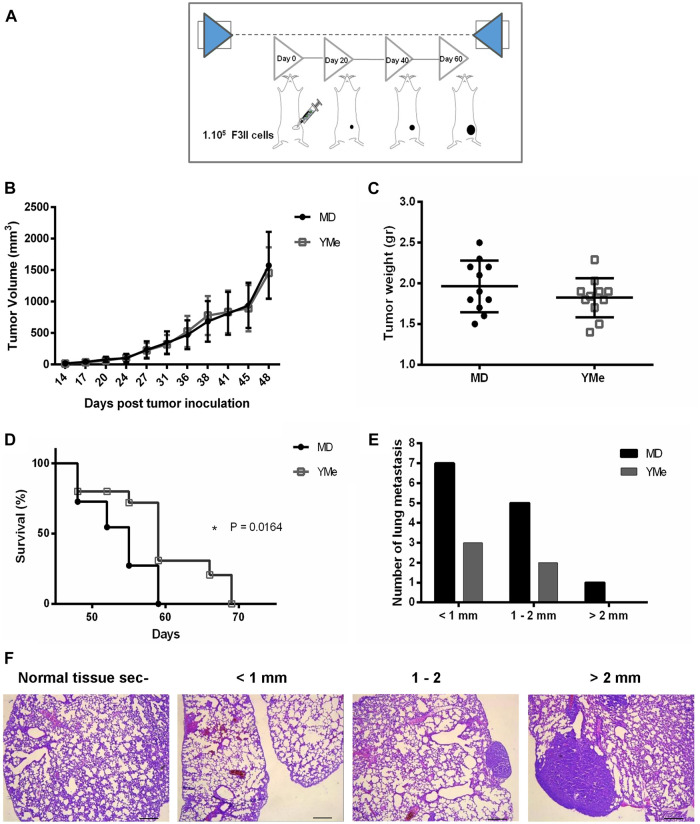
Effect of YMe on tumor progression in orthotopic BC model. BALB/c mice were randomly assigned into 2 groups (n = 11). Control group drank MD, the excipient of extract and treated group drank YMe 10 mg/ml. Both solutions were administered to the animals via the drinking water before (1 month) and after the inoculation of F3II tumor cells. The animals were inoculated subcutaneously with 1 × 10^5^ cells/mice on left mammary fat pad. At the end of the experiment, mice were sacrificed by cervical dislocation due to ethical considerations. **(A)** The timeline of the protocol is outlined in the scheme. **(B)** Tumor volumes, measured periodically with a caliper and calculated with the formula ½ (length x width^2^) (length > width), are expressed as the mean and analyzed using unpaired Student’s t test with Welch’s correction. **(C)** Tumor weight was registered. Unpaired Student’s t test was used to detect statistically significant differences. **(D)** Kaplan-Meier survival curves of YM-treated animals and controls. Statistical significance was calculated using log-rank test. **(E)** Number of lung metastasis based on size. The size of lung metastatic tumors was measured with a dissecting microscope and tumors were stratified into three groups based on size (diameter < 1 mm small size, 1–2 mm medium size and > 2mm, large size). The lung nodes of un-treated mice were increased in size compared with the nodes of treated mice. **(F)** Representative images of lung metastasis. Scale bars, 1 mm.

**FIGURE 6:**
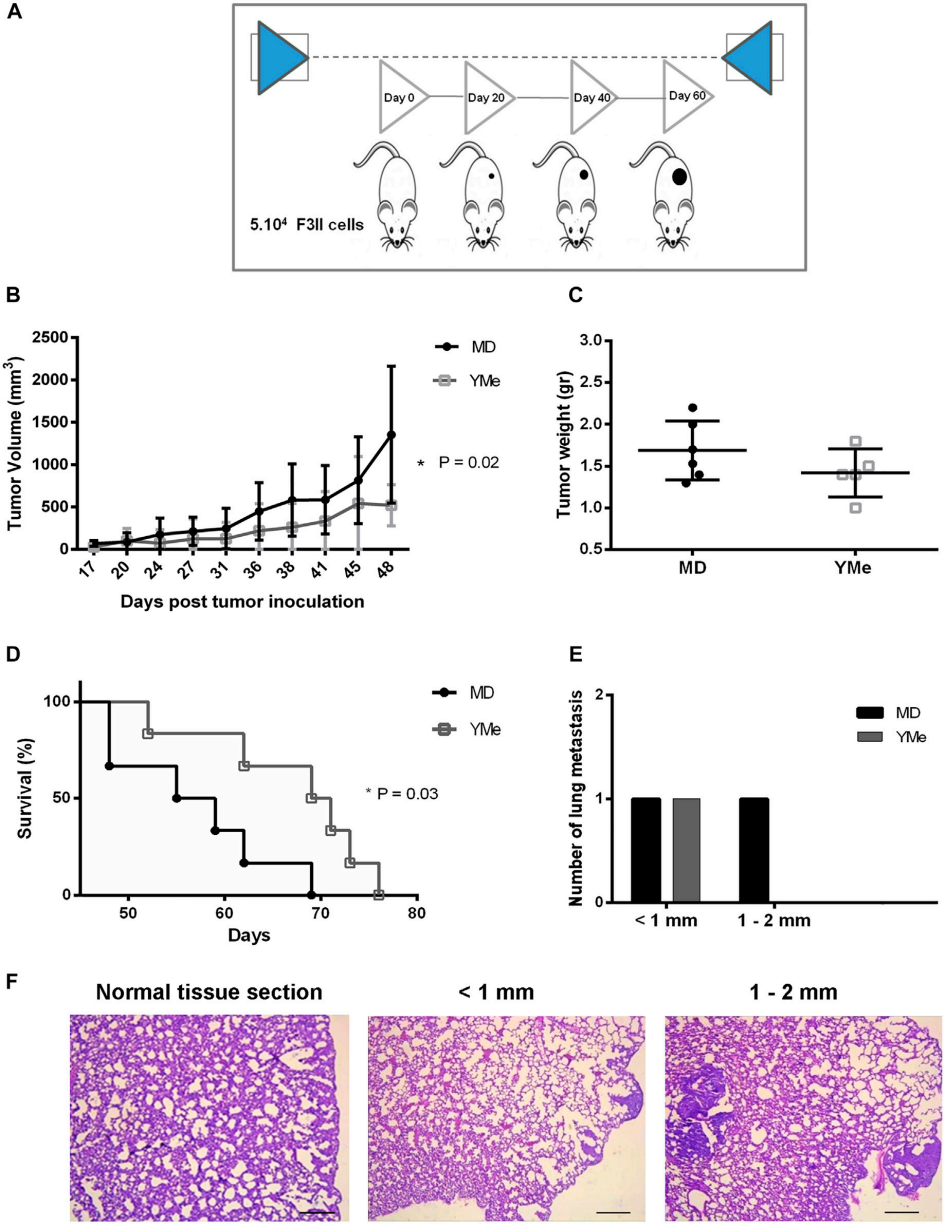
Effect of YMe on tumor progression in heterotopic BC model. BALB/c mice were randomly assigned into 2 groups (n = 6). Control group drank MD, the excipient of extract and treated group drank YMe 10 mg/ml. Both solutions were administered to the animal via the drinking water before (1 month) and after the inoculation of F3II tumor cells. The animals were inoculated subcutaneously with 5 × 10^4^ cells/mice on the right flank of female BALB/c mice. At the end of the experiment, mice were sacrificed by cervical dislocation due to ethical considerations. **(A)** The timeline of the protocol is outlined in the scheme. **(B)** Tumor volumes, measured periodically with a caliper and calculated with the formula ½ (length x width^2^) (length > width), are expressed as the mean and analyzed using unpaired Student’s t test with Welch’s correction. **(C)** Tumor weight was registered. Unpaired Student’s t test was used to detect statistically significant differences. **(D)** Kaplan-Meier survival curves of YM-treated animals and controls. Statistical significance was calculated using log-rank test. **(E)** Number of lung metastasis based on size. The size of lung metastatic tumors was measured with a dissecting microscope and tumors were stratified into three groups based on size (diameter < 1 mm small size, 1–2 mm medium size and > 2 mm, large size). **(F)** Representative images of lung metastasis. Scale bars, 1 mm.

The F3II cell/syngeneic mouse model was used to investigate the *in vivo* anti-tumor effects of YMe. Orthotopic and heterotopic BC models in BALB/c mice were used and the tumor progression was observed over time (Figures 5A, 6A respectively). In both models, the control group received a MD solution via the drinking water, while the treated group received YMe at a dose of 1.6 g/kg/day. Solutions were administered 1 month before the inoculation of F3II tumor cells and until the end of the protocol.

Throughout both studies, the body weight of mice in the control group and the YM group was monitored. Mice in all groups treated with MD or YMe gained weight progressively. As shown in Table 3, there were no significant differences in initial body weight, final body weight, body weight gain, and growth rate in all groups, *p* > 0.05 *vs*. control group. In addition, during the experiments, there were no signs of systemic toxicity, no behavioral abnormality, or animal death observed.

**TABLE 3:** Effects of YMe on different parameters in orthotopic and heterotopic *in vivo* breast cancer models.

*In vivo* model	Group	Initial body weigth (g)	Final body weigth (g)	Weigth gain (g)	Growth rate	Incidence of lung metastasis (%)
Orthotopic	MD	18.03 ± 0.92	24.52 ± 1.18	6.49 ± 1.26	0.09 ± 0.02	56
	YMe	17.04 ± 1.45^ns^	23.14 ± 2.05 ^ns^	6.10 ± 2.42	0.09 ± 0.03	28
Heterotopic	MD	16.52 ± 1.17	24.28 ± 1.53	7.76 ± 2.27	0.11 ± 0.03	33
	YMe	16.27 ± 1.54 ^ns^	23.57 ± 1.37 ^ns^	7.30 ± 2.53	0.10 ± 0.04	16

Weigth, ns: no significant difference between initial and final body weight among control and treated groups, *p* > 0.05 vs. control group (MD), Statistical analysis was done using Student’s t test.

Incidence of lung metastasis, defined as the number of mice with metastases related to the total number of inoculated ones was evaluated in both models. Statistical analysis was done using Mann-Whitney test. Orthotopic model *p* = 0.18 vs. control group. Heterotopic model *p* > 0.99 vs. control group.

The effect of YMe on the growth of orthotopic F3II primary tumors is shown in Figure 5B. The tumors first became palpable 7 days after injection and tumor take was 100% in groups 15 days after inoculation. By day 48, the mean tumor volume in both groups reached 1800 mm^3^; no statistically significant difference in mammary tumor volume between the two groups was observed.

The effect of YMe on the growth of heterotopic F3II primary tumors is shown in Figure 6B. The tumors became palpable 7 days after injection and tumor take was 100% in groups 15 days after inoculation. By day 48, we found that treatment with YMe significantly led to suppression of F3II tumor volumes when compared with the control group. Tumor volume in the control animals was the highest, reaching 1,355.29 ± 808.44 mm^3^ at the end of the experiment. In contrast, tumor volume in the YM group was significantly reduced, 521.34 ± 243.02 mm^3^. The oral administration of YMe at a dose of 1.6 g/kg/day resulted in a statistically significant decrease of tumor growth in the heterotopic model (*p* = 0.02).

At the end of the experiments, tumors were excised from each animal for examination of tumor weight and assessment of the anti-tumor effect of YMe. As shown in Figures 5C, 6C, both in the orthotopic model and in the heterotopic model respectively, we observed that tumor weight in the animals treated with YMe tended to be more reduced, although the differences compared with the control group were not statistically significant (*p* > 0.05). Unpaired Student’s t test was used for statistical analysis.

### Effect of YMe on Survival Time of Tumor-Bearing Mice

We further evaluated the effect of YMe on the survival time of F3II tumor-bearing mice. From day 48, the global survival analysis began in both models. It was observed that consumption of YMe significantly increased the survival of the animals (*p < 0.05) in both orthotopic and heterotopic models. As seen in Figure 5D, in the orthotopic model, the log-rank analysis showed that at day 59, 100% of the control group had been sacrificed while in the treated group only 62% of the experimental individuals had been sacrificed. As shown in Figure 6D, in the heterotopic model, the log-rank analysis indicate that at day 69, 100% of the control group had been sacrificed while in the group treated with the YMe only 50% of the experimental individuals had been sacrificed, which demonstrated that YMe prolongs the survival time of F3II tumor-bearing mice.

### Effect of YMe on Spontaneous Metastasis on Both Models

Also, we evaluated the effect of YMe on spontaneous lung metastasis. Lung tissues from mice of both BC models were dissected and fixed with Bouin’s solution to observe tumor metastasis. We could clearly see white nodules on the surfaces of lung lobes.

The incidence, (defined as the number of mice with metastases related to the total number of inoculated ones), is represented in Table 3. In the orthotopic model, only 28% of mice in the yerba mate group (3 of 11) had surface metastases, whereas that 56% of the mice in the control group (6 of 11) had surface lesions. However, the difference between the control and YM groups was not statistically significant (*p* > 0.05). The size of lung metastatic tumors was measured with a dissecting microscope and tumors were stratified into 3 groups based on size (diameter < 1 mm small size, 1–2 mm medium size and > 2 mm, large size) with the objective to do a qualitative analysis. As can be seen in Figure 5E, the size of lung nodules of un-treated mice was larger than to the ones of the animals treated with YMe. Interestingly, the pulmonary nodules in the mice treated with YMe did not exceed 2 mm.

The incidence of lung metastases in the heterotopic model is represented in Table 3. Whereas only 16% of mice in the YM group (1 of 6) had surface metastases, 33% of the mice in the control group (2 of 6) had surface lesions. The incidence of lung metastases was not statistically different between the control and Yerba Mate groups (*p* > 0.05). As Figure 6E shows, the number of medium lung metastases was reduced in treated mice compared to control animals. These results were further confirmed by H&E staining. Representative images of lung metastasis are shown in Figures 5F, 6F.

## Discussion

There is consensus that polyphenols act as anti-cancer agents by reducing cell growth, arresting cell cycle and inducing apoptosis (Rajamani 2018; Chaves et al., 2020; Dyshlovoy et al., 2020). In a previous work, our group showed that YMe, which contains a complex mixture of phytochemicals, reduces cell growth and induces apoptosis due to an activation of the intrinsic pathway in a colorectal cancer model. A significant decrease in Bcl-2 expression levels following YMe treatment (Garcia-Lazaro et al., 2020) was observed. We set out to discover whether the underlying mechanism of the effects of YMe on breast cancer cells was the same as the described in colon cancer. We demonstrated that YMe inhibited cell growth and increased cell apoptosis in BC models.

For the metastatic process to occur, the tumor cells must detach from the primary tumor and invade the surrounding tissue. A mechanism that plays a critical role in cancer metastasis is epithelial-mesenchymal transition (EMT), in which tumor cells convert an epithelial phenotype into a mesenchymal phenotype (Christofori 2006). These changes promote the loss of cell to cell adhesion and the gain of cell motility and invasiveness (Chambers et al., 2002; Thiery, 2002). We evaluated the effect of YMe on these events and demonstrated that YMe modulates cell migration and invasion on BC models. Polyphenols (bioactive compounds present in the extract) have anti-tumor activity as they can act on various molecular targets (Li et al., 2018). It is reported that curcumin and apigenin suppress cell migration and invasion by modulating the PI3K/Akt/mTOR signaling pathway in human glioblastoma cells (Maiti et al., 2019) and in a lung cancer model (Zhou et al., 2017) respectively. In addition, using an *in vitro* BC models researches demonstrated that mangiferin, a polyphenolic compound from Mangifera indica, and (-)-Epigallocatechin-3-gallate (EGCG), a polyphenolic compound from green tea, inhibit cell migration and invasion through Rac1 signalling (Deng, Tian, and Liang 2018; Y.; Zhang et al., 2009). Considering this knowledge, it is possible to hypothesize that YMe polyphenols act on certain actors of the signaling pathways involved in the processes of migration and cell invasion. However, further experiments are needed to corroborate this hypothesis.

*In vivo* syngeneic models are widely used tools to demonstrate activity of novel anti-cancer therapies. This type of models offers several advantages, one of which is that they allow the study of tumor tissue in an immunocompetent environment and of key steps in tumor progression such as angiogenesis, stromal-epithelial signaling, tissue invasion and metastasis.

The F3II cell/syngeneic mouse model was selected to investigate the *in vivo* anti-metastatic effect of YMe. F3II is a highly invasive and metastatic sarcomatoid mammary carcinoma cell line established from a clone of a spontaneous, hormone-independent BALB/c mouse mammary tumor (Alonso et al., 1996). Although there are many BC models to mimic the disease, the site of injection (subcutaneous or orthotopic into the fat pad of mouse) and the BC cell line used are crucial factors because they define both breast tumor progression and metastasis (Fantozzi and Christofori 2006). The site of the injection is key to determine the characteristics of the tumor microenvironment, which in turn will influence cell growth, metastatic ability and response to therapy. Orthotopic models mimic the location of the disease and the tumor microenvironment (Kocatürk and Versteeg 2015) while heterotopic models do not represent the local mammary tumor environment and the absence of this environment may result in BC development that differs from that observed in human pathology. This remarkable difference explains, in part, why the tumor growth curves of the two experimental models are totally different. In the heterotopic model, dietary intervention affects the growth ability of F3II cancer cells. However, under the influence of the microenvironment, YMe cannot reduce the growth rate with respect to the control group.

We also studied spontaneous metastasis in BC. The percentages of metastasis were different between models and treated groups. If we compare both control groups, we observed that the number of tumor metastases was higher in the orthotopic model than in the heterotopic model, 54 and 33% of tumor metastasis respectively. This difference is due to the fact that, in the orthotopic model, the cells from the primary tumor interact with the stromal microenvironment of the mammary gland which supports them and allows their growth and metastasis (Gout and Huot 2008). In contrast, the number of metastasis in the mice from the treated groups was 16% in the heterotopic model and 28% in the orthotopic. These results allow us to hypothesize that YMe modulates some steps of the metastatic cascade. Previously, we demonstrated the antiangiogenic potential of YMe *in vivo* using a subcutaneous angiogenesis assay in BALB/c mice (Garcia-Lazaro et al., 2020). In these BC models, we observed that tumors from treated mice in both models were smaller than tumors from the control group, which could be related to an underdeveloped vasculature. This allows us to suggest that the spread of tumor cells to non-contiguous organs is limited by YMe.

It is reported that polyphenols have an anti-metastatic *in vivo* effect and improve the survival of mice (Lee, 2009). As YMe is a source of these compounds, we evaluated the effect of the extract on this parameter. For the first time, we demonstrated that the chronic consumption of YMe had an impact on mice survival on both experimental BC models. This increase in survival may be related to the reduction of metastasis. It is necessary to highlight the biological relevance of this finding because this result was obtained by administering, via drinking water, an YMe at a dose of 1.6 g/kg/day. A particular emphasis is given on the composition of the extract; it has multiple active components which, conjugated at very low doses, could have a vastly effect.

Metastatic BC is one of the deadliest types of cancers worldwide in women (Tevaarwerk et al., 2014). Despite significant advances in both cancer diagnosis and treatment, most patients with advanced metastatic disease are unresponsive to current therapies. About 90% of cancer-associated deaths are estimated to be caused by metastatic disease rather than primary tumors (Lambert et al., 2018). Therefore, inhibition of the metastatic cascade could be a promising intervention in the clinical management of the disease. The results of our preclinical *in vitro* and *in vivo* studies suggest that the YMe could inhibit critical events related to metastatic spread; consequently, YMe would have a potential clinical application. However, further studies are necessary to confirm this hypothesis.

## Conclusion

The preclinical results in this work indicate that YM could be able to modulate key cellular functions during metastatic development. These findings suggest that YMe would have a potential role as an adjuvant in the clinical management of breast cancer.

## Data Availability

The original contributions presented in the study are included in the article/supplementary files, further inquiries can be directed to the corresponding author/s.

## References

[B1] Alonso D. F. Farías E. F. Urtreger A. Ladeda V. Vidal M. C. Bal De Kier Joffé E. (1996). Characterization of F3II, a Sarcomatoid Mammary Carcinoma Cell Line Originated from a Clonal Subpopulation of a Mouse Adenocarcinoma. J. Surg. Oncol. 62, 288–297. 10.1002/(SICI)1096-9098(199608)62:4<288:AID-JSO14>3.0.CO;2-1 8691844

[B2] Alves A. O. Weis G. C. C. Unfer T. C. Assmann C. E. Barbisan F. Azzolin V. F. (2019). Caffeinated Beverages Contribute to a More Efficient Inflammatory Response: Evidence from Human and Earthworm Immune Cells. Food Chem. Toxicol. 134, 110809. 10.1016/j.fct.2019.110809 31499124

[B3] Amigo-Benavent M. Wang S. Mateos R. Sarriá B. Bravo L. (2017). Antiproliferative and Cytotoxic Effects of Green Coffee and Yerba Mate Extracts, Their Main Hydroxycinnamic Acids, Methylxanthine and Metabolites in Different Human Cell Lines. Food Chem. Toxicol. 106, 125–138. 10.1016/j.fct.2017.05.019 28506698

[B4] Bastos D. H. M. Oliveira D. M. Matsumoto R. L. T. Carvalho P. O. Ribeiro M. L. (2007). Yerba Maté: Pharmacological Properties, Research and Biotechnology. Med. Aromatic Plant Sci. Biotechnol. 1 (1), 37–46.

[B5] Berté K. A. Beux M. R. SpadaSpada P. K. Salvador M. Hoffmann-Ribani R. (2011). Chemical Composition and Antioxidant Activity of Yerba-Mate (Ilex Paraguariensis A.St.-Hil., Aquifoliaceae) Extract as Obtained by Spray Drying. J. Agric. Food Chem. 59 (10), 5523–5527. 10.1021/jf2008343 21510640

[B6] Chambers A. F. Groom A. C. MacDonald I. C. (2002). Dissemination and Growth of Cancer Cells in Metastatic Sites. Nat. Rev. Cancer 2 (8), 563–572. 10.1038/nrc865 12154349

[B7] Chaves F. M. Pavan I. C. B. da Silva L. G. S. de Freitas L. B. Rostagno M. A. Antunes A. E. C. (2020). Pomegranate Juice and Peel Extracts Are Able to Inhibit Proliferation, Migration and Colony Formation of Prostate Cancer Cell Lines and Modulate the Akt/MTOR/S6K Signaling Pathway. Plant Foods Hum. Nutr. 75 (1), 54–62. 10.1007/s11130-019-00776-0 31838616

[B8] Christofori G. (2006). New Signals from the Invasive Front. Nature 441, 444–450. 10.1038/nature04872 16724056

[B9] Cragg G. M. Pezzuto J. M. (2016). Natural Products as a Vital Source for the Discovery of Cancer Chemotherapeutic and Chemopreventive Agents. Med. Princ Pract. 25 (2), 41–59. 10.1159/000443404 26679767 PMC5588531

[B10] da Veiga D. T. A. Bringhenti R. Copes R. Tatsch E. Moresco R. N. Comim F. V. (2018). Protective Effect of Yerba Mate Intake on the Cardiovascular System: a Post Hoc Analysis Study in Postmenopausal Women. Braz. J. Med. Biol. Res. 51, 1–5. 10.1590/1414-431X20187253 PMC5937722 29694507

[B11] Dai X. Ji Y. Jiang P. Sun X. (2017). Marsdenia Tenacissima Extract Suppresses Tumor Growth and Angiogenesis in A20 Mouse Lymphoma. Oncol. Lett. 13, 2897–2902. 10.3892/ol.2017.5831 28521395 PMC5431395

[B12] Deng Q. Tian Y. X. Liang J. (2018). Mangiferin Inhibits Cell Migration and Invasion through Rac1/WAVE2 Signalling in Breast Cancer. Cytotechnology 70 (2), 593–601. 10.1007/s10616-017-0140-1 29455393 PMC5851954

[B13] Dyshlovoy S. A. Tarbeeva D. Fedoreyev S. Busenbender T. Kaune M. Veselova M. (2020). Polyphenolic Compounds from Lespedeza Bicolor Root Bark Inhibit Progression of Human Prostate Cancer Cells via Induction of Apoptosis and Cell Cycle Arrest. Biomolecules 10. 10.3390/biom10030451 PMC7175281 32183314

[B14] Fantozzi A. Christofori G. (2006). Mouse Models of Breast Cancer Metastasis. Breast Cancer Res. 8, 212. 10.1186/bcr1530 16887003 PMC1779475

[B15] Filip R. Lotito S. B. Ferraro G. Fraga C. G. (2000). Antioxidant Activity of Ilex Paraguariensis and Related Species. Nutr. Res. 20 (10), 1437–1446. 10.1016/S0271-5317(00)80024-X

[B16] Garcia-Lazaro R. S. Lamdan H. Caligiuri L. G. Lorenzo N. Berengeno A. L. Ortega H. H. (2020). *In Vitro* and *In Vivo* Antitumor Activity of Yerba Mate Extract in Colon Cancer Models. J. Food Sci. 85 (7), 2186–2197. 10.1111/1750-3841.15169 32567699

[B17] Gout S. Huot J. (2008). Role of Cancer Microenvironment in Metastasis: Focus on Colon Cancer. Cancer Microenviron. 1, 69–83. 10.1007/s12307-008-0007-2 19308686 PMC2654352

[B18] Hanahan D. Weinberg R. A. (2011). Hallmarks of Cancer: The Next Generation. Cell 144 (5), 646–674. 10.1016/j.cell.2011.02.013 21376230

[B19] Heck C. I. De Mejia E. G. (2007). Yerba Mate Tea (Ilex Paraguariensis): A Comprehensive Review on Chemistry, Health Implications, and Technological Considerations. J. Food Sci. 72, R138–R151. 10.1111/j.1750-3841.2007.00535.x 18034743

[B20] Horwitz W. Latimer G. W. (2006). Official Methods of Analysis of AOAC International. Maryland: AOAC.

[B21] Kang N. J. Lee K. W. Kim B. H. Bode A. M. Lee H. J. Heo Y. S. (2011). Coffee Phenolic Phytochemicals Suppress colon Cancer Metastasis by Targeting MEK and TOPK. Carcinogenesis 32 (6), 921–928. 10.1093/carcin/bgr022 21317303 PMC3106432

[B22] Kapinova A. Kubatka P. Golubnitschaja O. Kello M. Zubor P. Solar P. (2018). Dietary Phytochemicals in Breast Cancer Research : Anticancer Effects and Potential Utility for Effective Chemoprevention. Environ. Health Prev. Med. 23, 36. 10.1186/s12199-018-0724-1 30092754 PMC6085646

[B23] Ko J. K. Auyeung K. K. (2013). Target-Oriented Mechanisms of Novel Herbal Therapeutics in the Chemotherapy of Gastrointestinal Cancer and Inflammation. Curr. Pharm. Des. 19 (1), 48. 10.2174/13816128130109 22950499

[B24] Kocatürk B. Versteeg H. H. (2015). Orthotopic Injection of Breast Cancer Cells into the Mammary Fat Pad of Mice to Study Tumor Growth. J. Vis. Exp. 96, 51967. 10.3791/51967 PMC4354624 25742185

[B25] Kou X. Han L. Li X. Xue Z. Zhou F. (2016). Antioxidant and Antitumor Effects and Immunomodulatory Activities of Crude and Purified Polyphenol Extract from Blueberries. Front. Chem. Sci. Eng. 10, 108–119. 10.1007/s11705-016-1553-7

[B26] Krishnan K. Khanna C. Helman L. J. (2006). The Molecular Biology of Pulmonary Metastasis. Thorac. Surg. Clin. 16, 115–124. 10.1016/j.thorsurg.2005.12.003 16805200

[B27] Lambert A. W. Pattabiraman D. R. Weinberg R. A. (2018). Emerging Biological Principles of Metastasis. Cell Emerging 168 (4), 670–691. 10.1016/j.cell.2016.11.037 PMC5308465 28187288

[B28] Lee S.-J. Chung I.-M. Kim M.-Y. Park K.-D. Park W.-W. Moon H.-I. (2009). Ill-Min Chung, Min-Young Kim, Keum-Duk Park, Won-Wan Park, and Hyung-In MoonInhibition of Lung Metastasis in Mice by Oligonol. Phytother. Res. 23 (7), 1043–1046. 10.1002/ptr.2810 19288502

[B29] Li S. Tan H. Y. Wang N. Cheung F. Hong M. Feng Y. (2018). The Potential and Action Mechanism of Polyphenols in the Treatment of Liver Diseases. Oxid. Med. Cell Longev. 2018, 1–25. 10.1155/2018/8394818 PMC5817364 29507653

[B30] Liu X. Du X. Han G. Gao W. (2017). Association between tea Consumption and Risk of Cognitive Disorders: A Dose-Response Meta-Analysis of Observational Studies. Oncotarget 8 (26), 43306–43321. 10.18632/oncotarget.17429 28496007 PMC5522147

[B31] Maiti P. Scott J. Sengupta D. Al-Gharaibeh A. Dunbar G. L. (2019). Curcumin and Solid Lipid Curcumin Particles Induce Autophagy, but Inhibit Mitophagy and the PI3K-Akt/MTOR Pathway in Cultured Glioblastoma Cells. Int. J. Mol. Sci. 20 (2), 399. 10.3390/ijms20020399 PMC6359162 30669284

[B32] Maqsood S. Benjakul S. Abushelaibi A. Alam A. (2014). Phenolic Compounds and Plant Phenolic Extracts as Natural Antioxidants in Prevention of Lipid Oxidation in Seafood: A Detailed Review. Compr. Rev. Food Sci. Food Saf. 13, 1125–1140. 10.1111/1541-4337.12106

[B33] Marventano S. Salomone F. Godos J. Pluchinotta F. Del Rio D. Mistretta A. (2016). Coffee and Tea Consumption in Relation with Non-alcoholic Fatty Liver and Metabolic Syndrome: A Systematic Review and Meta-Analysis of Observational Studies. Clin. Nutr. 35 (6), 1269–1281. 10.1016/j.clnu.2016.03.012 27060021

[B34] Niedzwiecki A. Roomi M. W. Kalinovsky T. Rath M. (2016). Anticancer Efficacy of Polyphenols and Their Combinations. Nutrients 8 (9), 552. 10.3390/nu8090552 PMC5037537 27618095

[B35] Pillar N. Polsky A. L. Weissglas-Volkov D. Shomron N. (2018). Comparison of Breast Cancer Metastasis Models Reveals a Possible Mechanism of Tumor Aggressiveness. Cell Death Dis. 9 (10), 1040. 10.1038/s41419-018-1094-8 30305609 PMC6180100

[B36] Rajamani K. Thirugnanasambandan S. S. (2018). Polyphenols from Brown Alga, Padina Boergesenii (Allendar & Kraft) Decelerates Renal Cancer Growth Involving Cell Cycle Arrest and Induction of Apoptosis in Renal Carcinoma Cells. Environ. Toxicol. 33, 1135–1142. 10.1002/tox.22619 30126067

[B37] Rates S. (2001). Plants as Source of Drugs. Toxicon Official J. Int. Soc. Toxinol. 39 (June), 603–613. 10.1016/S0041-0101(00)00154-9 11072038

[B38] Ronco A. L. Stefani E. D. Mendoza B. Vazquez A. Abbona E. Sanchez G. (2016). Mate and Tea Intake, Dietary Antioxidants and Risk of Breast Cancer: A Case-Control Study. Asian Pac. J. Cancer Prev. 17 (6), 2923–2933. 10.7314/apjcp.2016.17.3.1453 27356713

[B39] Scalbert A. Manach C. Morand C. Rémésy C. Jiménez L. Scalbert A. (2005). Dietary Polyphenols and the Prevention of Diseases. Crit. Rev. Food Sci. Nutr. 45, 287–306. 10.1080/1040869059096 16047496

[B40] Shi L. Yao H. Liu Z. Xu M. Tsung A. Wang Y. (2020). Endogenous PAD4 in Breast Cancer Cells Mediates Cancer Extracellular Chromatin Network Formation and Promotes Lung Metastasis. Mol. Cancer Res. 18, 735–747. 10.1158/1541-7786.MCR-19-0018 32193354 PMC7668292

[B41] Tevaarwerk A. J. Gray R. J. Schneider B. P. Smith M. L. Wagner L. I. Fetting J. H. (2014). Survival in Patients with Metastatic Recurrent Breast Cancer after Adjuvant Chemotherapy: Little Evidence of Improvement over the Past 30 Years. Cancer 119 (6), 1140–1148. 10.1002/cncr.27819.Survival PMC3593800 23065954

[B42] Thiery J. P. (2002). Epithelial-mesenchymal Transitions in Tumour Progression. Nat. Rev. Cancer 2 (June), 442–454. 10.1038/nrc822 12189386

[B43] Torre L. A. Siegel R. L. Ward E. M. Jemal A. (2016). Global Cancer Incidence and Mortality Rates and Trends-An Update. Cancer Epidemiol. Biomarkers Prev. 25 (January), 16–27. 10.1158/1055-9965.EPI-15-0578 26667886

[B44] Viguiliouk E. Kendall C. W. Blanco Mejia S. Cozma A. I. Ha V. Mirrahimi A. (2014). Effect of Tree Nuts on Glycemic Control in Diabetes: a Systematic Review and Meta-Analysis of Randomized Controlled Dietary Trials. PLoS One 9 (7), e103376. 10.1371/journal.pone.0103376 25076495 PMC4116170

[B45] Weisburger J. H. (2001). Chemopreventive Effects of cocoa Polyphenols on Chronic Diseases. Exp. Biol. Med. (Maywood) 226 (10), 891–897. 10.1177/153537020122601003 11682694

[B46] Yamagata K. Izawa Y. Onodera D. Tagami M. (2018). Chlorogenic Acid Regulates Apoptosis and Stem Cell Marker-Related Gene Expression in A549 Human Lung Cancer Cells. Mol. Cel Biochem. 441 (1), 9–19. 10.1007/s11010-017-3171-1 28875417

[B47] Yates L. R. Knappskog S. Wedge D. Farmery J. H. R. Gonzalez Campbell S. Martincorena I. (2017). Genomic Evolution of Breast Cancer Metastasis and Relapse. Cancer Cell 32, 169–184. 10.1016/j.ccell.2017.07.005 28810143 PMC5559645

[B48] Yoon J.-H. Baek S. J. (2005). Molecular Targets of Dietary Polyphenols with Anti-inflammatory Properties. Yonsei Med. J. 46 (5), 585–596. 10.3349/ymj.2005.46.5.585 16259055 PMC2562783

[B49] Zhang H. Tsao R. (2016). Dietary Polyphenols, Oxidative Stress and Antioxidant and Anti-inflammatory Effects. Curr. Opin. Food Sci. 8, 33–42. 10.1016/j.cofs.2016.02.002

[B50] Zhang Y. Han G. Fan B. Zhou Y. Zhou X. Wei L. (2009). Green tea (-)-Epigallocatechin-3-Gallate Down-Regulates VASP Expression and Inhibits Breast Cancer Cell Migration and Invasion by Attenuating Rac1 Activity. Eur. J. Pharmacol. 606 (1), 172–179. 10.1016/j.ejphar.2008.12.033 19171136

[B51] Zhou Z. Tang M. Liu Y. Zhang Z. Lu R. Lu J. (2017). Apigenin Inhibits Cell Proliferation, Migration, and Invasion by Targeting Akt in the A549 Human Lung Cancer Cell Line. Anticancer Drugs 28 (4), 446–456. 10.1097/CAD.0000000000000479 28125432

